# Internet and Telerehabilitation-Delivered Management of Rotator Cuff–Related Shoulder Pain (INTEL Trial): Randomized Controlled Pilot and Feasibility Trial

**DOI:** 10.2196/24311

**Published:** 2020-11-18

**Authors:** Peter Malliaras, Kate Cridland, Ruben Hopmans, Simon Ashton, Chris Littlewood, Richard Page, Ian Harris, Helen Skouteris, Terry Haines

**Affiliations:** 1 Physiotherapy Department, School of Primary and Allied Health Care, Faculty of Medicine Nursing and Health Science Monash University Melbourne Australia; 2 Faculty of Health, Psychology and Social Care Manchester Metropolitan University Manchester United Kingdom; 3 Barwon Orthopaedic Research and Education, Barwon Health and School of Medicine Deakin University Geelong Australia; 4 Whitlam Orthopaedic Research Centre, Ingham Institute for Applied Medical Research Sydney Australia; 5 South Western Sydney Clinical School University of New South Wales Sydney Australia; 6 Liverpool Hospital Sydney Australia; 7 Monash Centre for Health Research and Implementation, School of Public Health and Preventive Medicine Monash University Melbourne Australia; 8 School of Primary and Allied Health Care, Faculty of Medicine Nursing and Health Science Monash University Melbourne Australia

**Keywords:** rotator cuff, tendinopathy, shoulder, telemedicine, telerehabilitation, randomized controlled trial, pilot, feasibility, pain, internet-delivered intervention

## Abstract

**Background:**

Rotator cuff–related shoulder pain (RCRSP) is a common and disabling musculoskeletal condition. Internet-based and telerehabilitation delivery of recommended care may improve access to care and improve adherence and outcomes.

**Objective:**

The primary aim of this pilot randomized controlled trial was to assess the feasibility of a 12-week internet-delivered intervention for RCRSP comparing advice only, recommended care, and recommended care with group-based telerehabilitation.

**Methods:**

Reporting was in accordance with the Consolidated Standards of Reporting Trials (CONSORT) checklist for pilot and feasibility trials. People with a primary complaint of RCRSP for 3 months or longer were identified via a paid Facebook strategy. Screening involved an online questionnaire followed by a 20-minute telehealth assessment. Participants were randomly allocated (via a Zelen design) to receive (1) advice only, (2) recommended care (internet-delivered evidence-based exercise and education), or (3) recommended care and telerehabilitation (including a weekly group teleconference session). Progression criteria for a full-scale trial included (1) recruitment of 20% or greater of eligible participants, (2) acceptable adherence (two or more of the three prescribed weekly sessions) among 70% or greater of participants, (3) 80% or greater retention of participants, (4) absence of intervention-related serious adverse events, and (5) 80% or greater response rates to questionnaires. Secondary clinical and patient knowledge outcomes were collected (via email or text) at baseline, six weeks, and 12 weeks (for clinical and patient knowledge), and within-group change was reported descriptively.

**Results:**

We enrolled 36 of 38 (95%) eligible participants and all participants were recruited within a 3-week period. Of the 36 participants, 12 participants were allocated to each of the three trial arms. The mean age of participants was between 51 and 56 years, and 83% (10/12) to 92% (11/12) were female. Retention at the 12-week endpoint was 94% (34/36) and response to email questionnaires at other time points was 83% or greater. We found acceptable adherence (defined as greater than 70% of participants performing exercise 2 or 3 times/week) in the recommended care group with telerehabilitation but not in the recommended care group without telerehabilitation. There was a total of 24 adverse events over 108 person-months of observation. All adverse events were mild or moderate (mainly muscle and shoulder symptoms), with the exception of one instance of elective surgery (unrelated to the person’s shoulder condition).

**Conclusions:**

Our prespecified success criteria were met or exceeded, but there was a gender imbalance toward women. It is feasible to progress to a fully powered trial, but strategies to address the gender imbalance need to be implemented.

**Trial Registration:**

Australian New Zealand Clinical Trials Registry (ACTRN12620000248965); https://tinyurl.com/yy6eztf5

## Introduction

Rotator cuff–related shoulder pain (RCRSP) is a common and disabling musculoskeletal condition. The estimated point prevalence of shoulder pain among adults is between 15% and 27% [1], and RCRSP is regarded as the most common cause, accounting for 70% of cases [2]. RCRSP can severely limit work and daily functions, including dressing and personal care, and can lead to substantial societal burden through utilization of health care resources and work absenteeism [1,3]. Up to 40% of those affected experience ongoing pain and disability beyond 12 months and many eventually require further interventional care (eg, injection, surgery) [1]. The estimated annual cost of managing shoulder pain is US $5,234.54 per person in 2009, and this is heavily influenced by sick leave and surgical costs [4].

Clinical practice guidelines recommend up to 12 weeks of conservative care (activity, medication, and exercise) for first presentation of RCRSP prior to considering imaging or surgery [5-7]. In contrast, high rates of imaging for first presentation of RCRSP were observed in a general practitioner (GP) database study (55%) [8] and a survey of GPs (82%) [9], both undertaken in Australia. One in 6 GPs would also refer for surgical opinion [9]. These imaging and surgical referral practices involve significant costs and, in some cases, unnecessary surgery [10], and may partly explain the doubling of rotator cuff–related surgeries and tripling of associated costs in Western Australia in just over a decade (2001-2013) [11].

Although the decision is multifactorial, one reason clinicians provide care that is not guideline recommended is to appease patients [12,13]. Pressure from patients for imaging and surgical referrals may stem from beliefs about the relevance of pathoanatomy and imaging findings [14,15]. Further, patients’ beliefs about expected outcomes are strong predictors of conservative care outcomes for shoulder pain [16] and RCRSP [17]. Educating patients directly about their condition and recommended care has the potential to improve health literacy, quality of care, and health outcomes [18-20]. Additionally, patient-directed education circumvents clinician-related barriers to recommended care that can be challenging to influence.

By increasing access to guideline-recommended care, internet-based delivery of patient-directed recommended care may improve quality of care and outcomes. Internet-based delivery of patient-direct recommended care is convenient and enables care delivery to people in rural and remote regions [21-23]. However, internet delivery of health care (eg, for low back pain and chronic pain) has demonstrated heterogeneous effects on health care use and clinical outcomes [24,25]. Telerehabilitation may improve outcomes of internet-based care delivery [26] and reduce attrition [27] and may be able to replace face-to-face care that includes exercise [21] while reducing costs [28]. Further, group-based telerehabilitation may be more cost-effective and includes an opportunity for peer-to-peer support, and its outcomes are comparable with those for individual care for musculoskeletal conditions [29]. Internet-based delivery of care with or without telerehabilitation has the potential to improve quality of care and outcomes for people with RCRSP, but it is not known whether investigating these interventions in RCRSP is feasible.

The primary aim of this study was to assess the feasibility of a future substantive randomized controlled trial (RCT) comparing the effectiveness of three internet-delivered interventions for RCRSP (advice only, recommended care, and recommended care with telerehabilitation). Primary feasibility aims included assessing (1) rates of conversion and recruitment, (2) levels of adherence, (3) rate of retention, (4) incidence of adverse events, and (5) response rates to questionnaires. A secondary aim was to explore any signals of treatment effect and variability in clinical outcomes at 6 and 12 weeks.

## Methods

### Study Design

The study was a 3-arm, parallel-group pilot and feasibility RCT. Study design and reporting were in accordance with the Consolidated Standards of Reporting Trials (CONSORT) eHealth guidelines [30] and extension for randomized pilot and feasibility trials [31], as well as the Consensus on Exercise Reporting Template (CERT) [32]. The trial protocol was prospectively registered on February 26, 2020, at the Australian New Zealand Clinical Trials Registry (ACTRN12620000248965). Ethical approval was granted by the Monash University Human Ethics Committee (No. 22338).

### Recruitment Strategy and Incentive

Thirty-six community-dwelling people with RCRSP were recruited and randomized into 3 groups (12 individuals per group). Recruitment was via a paid Facebook campaign. To compensate them for their time, participants were incentivized by receiving an Aus $100 (US $70) shopping voucher on completion of their 12-week questionnaire (regardless of other outcome data returned). This strategy was considered important to improve participant retention [33-35].

### Internet Eligibility Screening

Inclusion was based on the following question set (they were excluded if they answered one or more questions with the response indicated in parenthesis): (1) Has your shoulder problem been diagnosed by a health professional as frozen shoulder, arthritis, a labral tear, instability? (yes), (2) Is your shoulder pain a result of a shoulder dislocation? (yes), (3) Is your shoulder pain mainly around the area shown in the photos (anterolateral upper arm/shoulder pain)? (no), (4) Is your shoulder pain made worse by neck movement? (yes), (5) Is your shoulder pain brought on by moving your arm above your head? (no), and (6) Are you able to lift your arm to the height in the photo (90 degrees of elevation)? (no). The last question was designed to exclude people with massive rotator cuff tears involving multiple tendons or frozen shoulder [7]. People who were younger than 18 years, had shoulder pain for less than 3 months, had had prior surgery for their currently most symptomatic shoulder, or who had another complaint more troubling than their shoulder were excluded. People were also excluded if they indicated that they were severely depressed; taking recreational drugs, oral steroids (eg, prednisolone), or blood thinning medications (eg, warfarin); had angina, heart problems, or severe middle abdominal or upper back pain; had a history of cancer; had recent dizziness, blurred vision, slurred speech, difficulty swallowing, falls, or unsteadiness; or had a recent seizure. People excluded were advised to seek advice from their GP.

### Telerehabilitation Eligibility Screening

We undertook a 20-minute teleconference (Zoom) session with participants to check answers to questions 1 to 6 described above (regardless of answers to these questions). This was only done with people who passed the remaining online screening questions.

### Randomization

Participants who passed both screening stages completed an electronic consent form and were then eligible to be randomized. We utilized the two-stage Zelen randomization process in order to minimize refusal to participate related to randomization and reduce attrition (which is relatively common in the context of internet-based interventions) related to resentful demoralization in the active control group. Eligible participants were initially informed and consented to participate in a single-cohort study involving the advice-only group. The cohort was then randomized into the three groups. People randomized to one of the two intervention groups were invited to join their allocated group and were provided with information about the recommended care provided in these groups. If they agreed, they completed a second electronic consent form specific to their group allocation. Randomization was via a computer-generated random number sequence. To ensure allocation concealment, a researcher who had no contact or knowledge of any characteristics of the participants performed the randomization. Once participants were screened, the independent researcher accessed the trial database and randomized the 36 included participants. The same researcher then initiated (via emails to the participants) treatment for people in the advice-only and recommended care groups, and allocated (randomly) 3 participants to each of the 4 physiotherapists providing care in the recommended care and telerehabilitation group.

### Blinding

The trial was single-blind (assessors), as all outcomes were collected via self-report questionnaires. Clinicians providing the telerehabilitation intervention were aware of treatment allocation. Because of the Zelen design, all participants perceived receiving an intervention, and participants in the recommended care groups knew their intervention contained additional components.

### Baseline Assessment

Baseline assessment was via an online questionnaire and included demographic factors, medical history, site and duration of symptoms, previous treatments, and baseline outcomes (described below).

### Internet Intervention Content Development

Content was based on available guidelines, systematic reviews and consensus statements [5-7,36-41]. In developing the education intervention, we also sought the views of 8 international clinical shoulder researchers (semistructured interviews) [42] and 8 patients with RCRSP (focus group).

### Internet Intervention Format and Testing

Interventions included text, infographics, and videos (created with Powtoon animation software; Powtoon Ltd). Following development of the content for each of the groups, the international clinical shoulder researchers and patients were invited to provide feedback regarding the accuracy, adequacy, and clarity of the content. The Patient Education Materials Assessment Tool (PEMAT) was used to assess understandability and actionability of the multimedia education content [43]. Minor changes were made (layout and wording) based on clinician and patient feedback and the PEMAT results.

### Internet-Based Interventions

Each of the internet-based interventions was 12 weeks in duration.

#### Advice Only

The advice-only group received education about the rotator cuff muscles and risk factors (Multimedia Appendix 1) and advice about modifying general and work-related activities (Multimedia Appendix 2) consistent with current guidelines [5,6]. Activity modification was labelled an intervention so that participants perceived that they were receiving treatment. Participants were advised to continue day-to-day and work activities if pain during these tasks was “acceptable” (a score of 4 or less on an 11-point numerical rating scale, with 10 representing worst pain imaginable) [44]. It was also acceptable for pain after activity to increase if it returned to pre-exercise levels within a reasonable period [44]. Participants were advised to stop or modify (ie, reduce volume of) activities until they could be performed with acceptable pain.

#### Advice With Recommended Care

Recommended care included exercise (described below) and education about the causes of RCRSP and pain mechanisms, as well as education about exercise and other treatments and their effectiveness and potential harms (Multimedia Appendix 3). Emphasis was on addressing knowledge gaps and barriers and enablers to recommended care. For example, challenging participants’ understanding of the relevance of tendon structure and imaging [15], expectations from exercise interventions, and pain and exercise self-efficacy [16]. The education intervention was informed by adult learning theory [45] and evidenced-based principles of self-management and cognitive behavioral therapy [46].

#### Advice With Recommended Care and Telerehabilitation

The group receiving recommended care and telerehabilitation was identical to the recommended care group, but it also received a weekly telerehabilitation session with a physiotherapist via free videoconferencing software (Zoom; Zoom Video Communications Inc). Participants were provided with information about how to set up the telerehabilitation environment, including how to position their phone, tablet, or laptop (desktops were discouraged), video settings, and camera angle. Prior to starting, the telerehabilitation environment was extensively piloted for this context. In the first two rehabilitation sessions (60 minutes each), the physiotherapists presented PowerPoint slides (screensharing via Zoom) providing education about RCRSP (Session 1: Understanding RCRSP and the treatments available; Session 2: Exercise and self-management). In the remaining sessions (30 minutes each), the physiotherapists prompted discussion about pain and pathology beliefs, exercise expectations, and individual physical activity goals, as well as monitoring and providing feedback regarding exercise fidelity.

### Exercise Intervention

**Figure 1:**
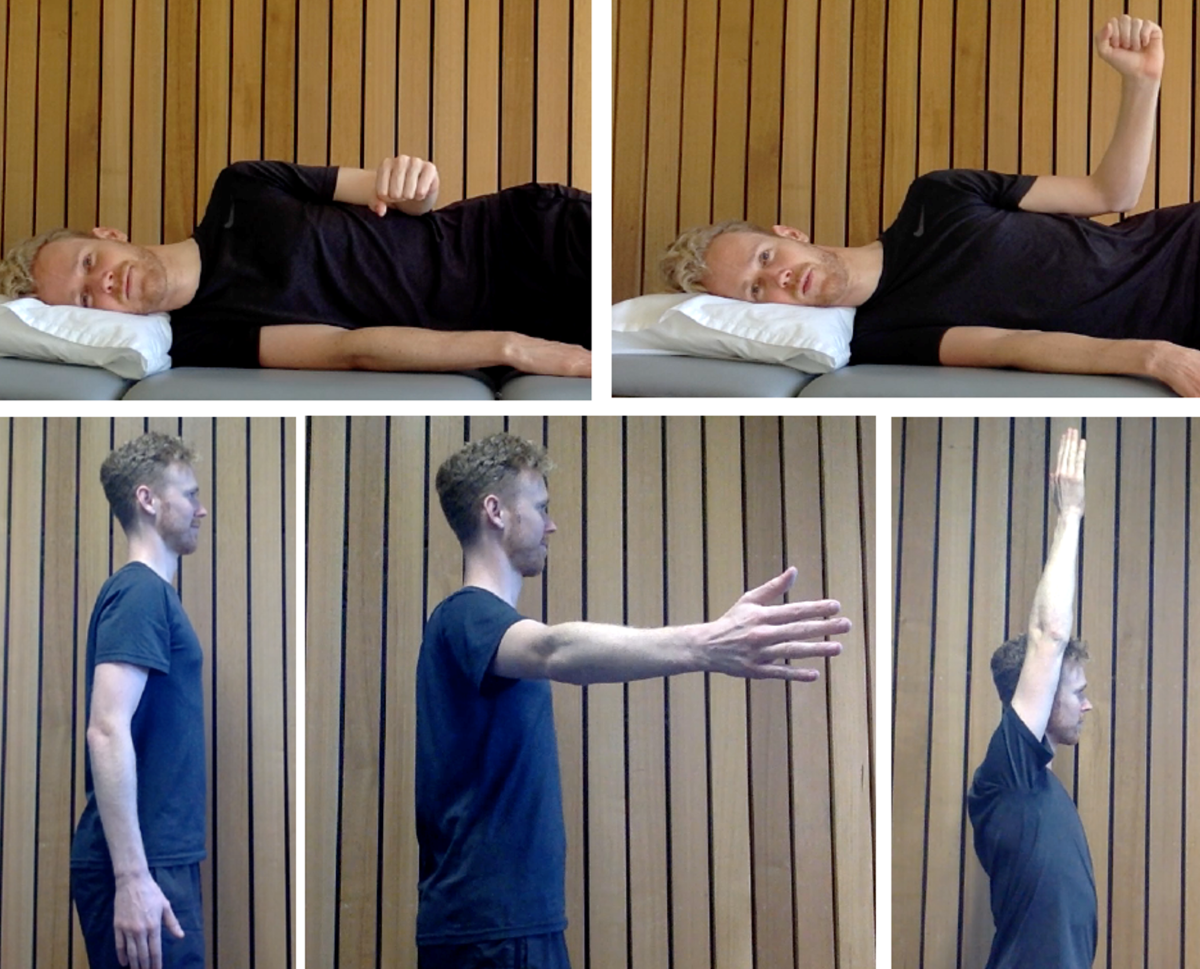
External rotation and elevation exercises.

The exercise approach was identical for the recommended care and recommended care and telerehabilitation groups and was based on available evidence and expert consensus [37-40,47]. Two exercises were included (Figure 1): shoulder elevation in standing position from 10 to 150 degrees, and external rotation in side-lying position, full range. Elevation was performed as shoulder abduction, scapular plane elevation or flexion, depending on acceptable pain response (as defined above) and patients’ preference. Progression and regression were based on pain or how difficult the exercise was (Multimedia Appendix 4). If pain during exercise was 5 or greater on the 11-point numerical rating scale or the exercise was too difficult (allocated repetitions could not be completed), the following modifications were trialed (in this order): (1) reduce load if participant was using a weight, (2) reduce range of motion and/or the number of repetitions, or (3) revert to isometric hold exercise. This process was reversed to progress exercise when the pain was acceptable or the allocated volume did not achieve muscular fatigue (defined below). Participants were instructed to perform the exercises 3 times per week for 12 weeks: 3 sets of 15 repetitions and 4 seconds per cycle for isotonic exercises (2-second concentric and 2-second eccentric phase) or 6 sets of 30 seconds for isometric exercise, with a 2-minute rest between each set. Fatigue was defined as self-reported inability to complete a further complete repetition. Participants were encouraged to use a heavier weight if they felt they could do 17 repetitions or more, or a lighter weight if they could do 13 repetitions or fewer). Participants were advised to adjust the weight in 0.5 kg or 1 kg increments and use their own dumbbells at home or an empty container (eg, large plastic milk container) filled with water until it approximated the desired weight. Participants entered pain and fatigue data onto the website (Multimedia Appendix 5) prior to commencing the exercise session and after each set of exercise, and the algorithm provided guidance on progression and regression.

### Behavioral Strategies for Intervention Groups

Strategies to improve adherence included an interactive website-based exercise progression and regression tool with built-in electronic exercise diary (Multimedia Appendix 5), allowing patients some choice in exercise selection (ie, elevation plane), challenging cognitions and beliefs that might impact adherence (eg, pain beliefs or fear avoidance, as described above), and providing regular feedback and monitoring (telerehabilitation group) [48].

### Physiotherapist Recruitment and Training

Four registered physiotherapists working in primary care settings with experience managing shoulder conditions were recruited to deliver the telerehabilitation interventions. The physiotherapists participated in four 1-hour training sessions via teleconference to learn about the intervention and standardize the delivery of care.

### Primary Feasibility Outcomes

The following outcomes (based on a high-quality trial undertaken in the same geographical region and using similar recruitment strategies [49]) were used to determine feasibility (ie, yes, no, or modifications required) for a substantive RCT:

- the number of eligible participants who consented and were randomized (feasible defined as 20% or greater).
- exercise adherence—participants were asked whether they completed 0, 1, 2, or 3 or more of the 3 prescribed sessions per week; acceptable adherence was defined as 70% or more participants in a group completing 2 or more prescribed exercise sessions) per week.
- rate of retention (feasible defined as 80% or greater).
- response rates to questionnaire outcomes (feasible defined as 80% or greater).
- incidence of adverse events—adverse events were defined as any symptom experienced during the trial that may or may not be causally related to the intervention; the frequency (number of participants and number of adverse events), type (eg, muscle soreness), and severity (mild: lasting less than 7 consecutive days; moderate: lasting 7 consecutive days or longer; severe: results in death, life-threatening complication, hospitalization, surgery, permanent or temporary physical disability, congenital abnormalities, or any findings the research team believed could lead to significant health hazards).

Adverse events and adherence were reported via email questionnaire at 4, 8, and 12 weeks.

### Outcomes for Economic Evaluation

Outcomes for economic evaluation were included to assess the feasibility of collection in a future full-scale trial (feasible defined as a response rate of 80% or greater). Health care use related to RCRSP was assessed with a patient questionnaire. Productivity (including absenteeism and presenteeism) was measured using the iMTA Productivity Cost Questionnaire [50]. These outcomes were collected via email questionnaire at 4, 8, and 12 weeks. Costs were divided into direct costs (intervention related and other) and indirect costs (including absenteeism and presenteeism). Physiotherapists’ hourly rate was assumed to be Aus $150 (US $106) and average Australian hourly rate to be Aus $60 (US $42).

### Secondary Clinical Outcomes

Clinical outcomes were assessed at baseline, 6 weeks, and 12 weeks via email questionnaire as follows:

- pain and function—measured with the Shoulder Pain and Disability Index (SPADI), a validated questionnaire [51] that has been used extensively among people with RCRSP; the minimal clinically important difference (MCID) for SPADI among people with RCRSP is reported to be between 8 and 13 points [52].
- global rating of change—measured using the patient Global Rating of Change (GROC) 11-point Likert scale [53]; participants were asked to rate how their shoulder pain had changed since receiving the intervention.
- worst pain in the last 7 days—measured using the 100-mm visual analog scale (VAS; zero=no pain, 100=worst pain possible).
- health-related quality of life—measured with the EuroQol 5D-5L (EQ5D), a validated and reliable tool [54], including 5 domains (mobility, self-care, usual activities, pain/discomfort, and anxiety/depression).
- kinesiophobia (fear of movement)—measured with the Tampa Scale for Kinesiophobia (TSK), which has been validated among people with musculoskeletal pain [55].
- pain catastrophizing—measured with the Pain Catastrophizing Scale (PCS), a validated measure of pain catastrophizing [56].
- pain self-efficacy—measured with the reliable, valid, and responsive short form of the Pain Self-Efficacy Questionnaire (PSEQ) [57].
- RCRSP knowledge—assessed with a custom knowledge test at 0 (pre- and immediately post-education), 6, and 12 weeks. The questionnaire was developed based on the patient knowledge questionnaire for patients with osteoarthritis (OA) [58] and included 16 multiple-choice questions (6 questions about the disease, eg, risks and symptoms, and 10 questions about treatments, and treatment efficacies and harms). Psychometric properties of this scale were evaluated and deemed acceptable.

If participants did not complete email questionnaires within 7 days, they were reminded via email (2 emails/week) and later via a phone call.

### Statistical Analysis

Data were analyzed using SPSS (version 25; IBM Corp). Frequencies and proportions were found for categorical data, means and SDs for continuous data, and medians and IQRs for ordinal data. This pilot RCT was not powered to detect comparative treatment effects; however, within-group mean differences and 95% CIs as well as within-group standardized mean differences (SMDs, mean difference/pooled SD) were reported for change between baseline and 6 weeks and between baseline and 12 weeks. The SMD was interpreted in the following way: 0.2=small effect, 0.5=moderate effect, 0.8=large effect, and 1.2=very large effect [59]. The GROC was dichotomized, where a successful outcome was defined as “moderately better,” “much better,” or “very much better” on this scale [53].

## Results

Demographic data for the 36 participants recruited are shown in Table 1. The mean age of participants was 53.9 (SD 12.0) years and 89% (32/36) of the cohort was female.

**Table 1:** Demographic information of study participants.

Demographic factor		Advice only (n=12)	Recommended care (n=12)	Recommended care and telerehabilitation (n=12)
Age (years), mean (SD)		53.7 (11.5)	51.3 (13.7)	56.6 (11.0)
Female, n (%)		11 (92)	10 (83)	11 (92)
Height (cm), mean (SD)		166.3 (7.0)	170.8 (11.8)	165.1 (8.2)
Mass (kg), mean (SD)		84.3 (24.7)	74.9 (16.9)	76.4 (12.6)
BMI (kg/m²), mean (SD)		30.9 (10.3)	25.9 (5.7)	28.0 (4.2)
Employed, n (%)		6 (50)	8 (67)	5 (42)
**Residence, n (%)**				
	Urban	7 (58)	9 (75)	9 (75)
	Other urban	4 (25)	2 (17)	3 (25)
	Rural	1 (8)	1 (8)	0 (0)
Affected/worst side is right side, n (%)		8 (67)	6 (50)	7 (58)
Duration of symptoms (weeks), mean (SD)		27.6 (17.1)	42.5 (17.7)	33.8 (20.0)
Prior exercise treatment, n (%)		8 (67)	8 (67)	9 (75)
Prior activity modification, n (%)		2 (17)	5 (42)	6 (50)
Prior shoulder imaging, n (%)		3 (25)	6 (50)	3 (25)
SPADI, mean (SD)		30.6 (17.7)	37.3 (16.7)	41.8 (19.0)
Worst pain in the previous week, mean (SD)		5.4 (2.8)	7.5 (2.0)	6.3 (3.8)
**Comorbidities, n (%)**				
	Osteoarthritis	5 (42)	0 (0)	3 (25)
	Rheumatoid arthritis	0 (0)	2 (17)	0 (0)
	Inflammatory bowel disease	1 (8)	0 (0)	0 (0)
	Fibromyalgia	0 (0)	2 (17)	0 (0)
	Hypertension	1 (8)	0 (0)	1 (8)
	Hypercholesterolemia	0 (0)	2 (17)	5 (42)
	Diabetes	1 (8)	1 (8)	3 (25)

### Primary Feasibility Outcomes

#### Rate of Conversion and Recruitment

**Figure 2:**
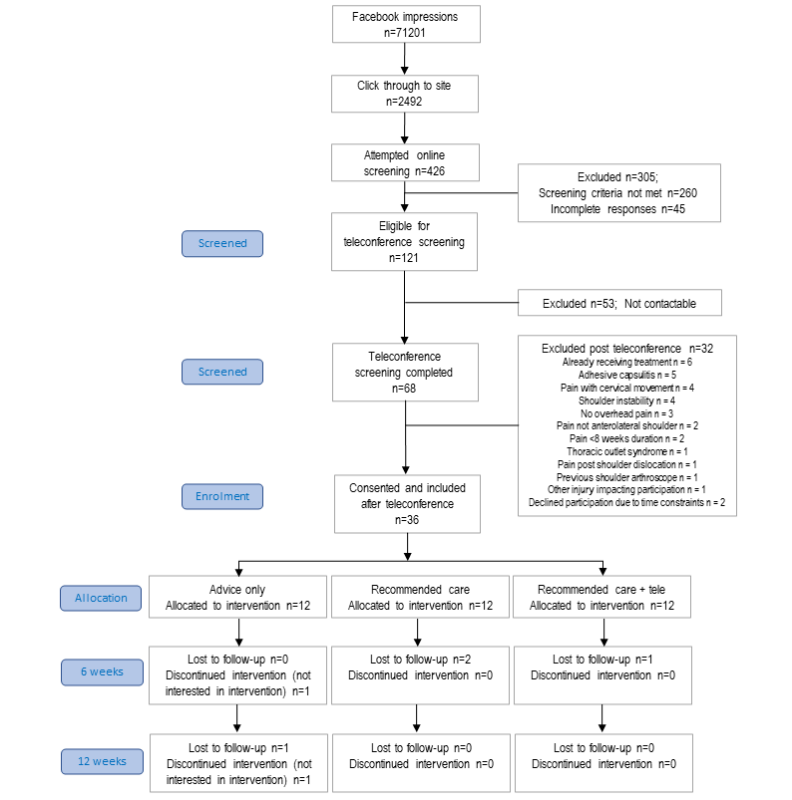
Consolidated Standards of Reporting Trials (CONSORT) flow diagram. tele: telerehabilitation.

The CONSORT flow diagram is shown in Figure 2. The total cost of the Facebook advertising campaign was Aus $2,146.02 (US $1512.94). The campaign was active for 21 days over 4 weeks—average cost of Aus $59.61 (US $42.03) per participant recruited)—which generated 71,201 impressions and 2492 clicks to the trial site. There were 68 potentially eligible participants who were screened via teleconference, of which 38 were eligible and 36 consented to participate (rate of conversion=36/38=95% of eligible participants recruited). All 36 participants were recruited in 1 calendar month.

#### Retention and Email Questionnaire Response Rates

The questionnaire data at 12 weeks were submitted via email by 34 participants (34/36, 94% retention). Response to email questionnaires at 4 weeks (32/36, 88%), 6 weeks (32/36, 88%), and 8 weeks (30/36, 83%) were also acceptable.

#### Exercise and Telerehabilitation Adherence

**Figure 3:**
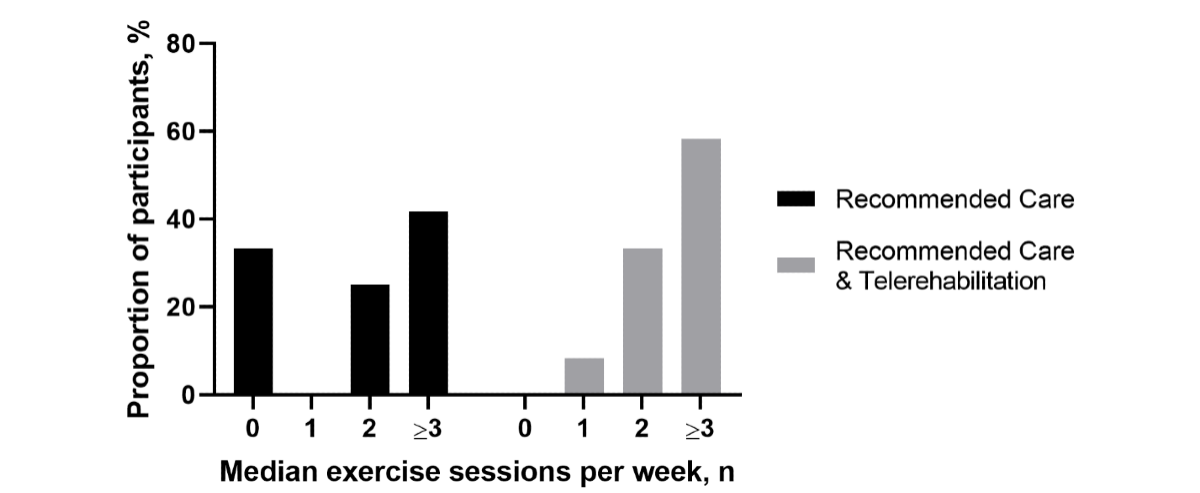
Exercise adherence in patients receiving recommended care and recommended care with telerehabilitation.

Acceptable exercise adherence was achieved in the recommended care group (11/12, 92%) but not without telerehabilitation (8/12, 67%) (Figure 3). One-third (4/12, 33%) of people in the recommended care without telerehabilitation group performed no exercise at all. A mean of 10.6 (SD 1.4) out of 12 (88%) teleconference sessions were attended.

#### Adverse Events

Adverse events reported were classified as either mild or moderate (Table 2). There was 1 serious adverse event—a participant in the recommended care with telerehabilitation group had elective surgery (brain surgery unrelated to RCRSP) and was absent from the trial for approximately 3 weeks. Other adverse events in all 3 groups could be categorized as either increased shoulder symptoms or muscle soreness around the shoulder, with the exception of 2 incidences of back pain in the advice-only group.

#### Co-interventions

Two people (2/12, 17%) in each group reported between 14 and 16 incidences of co-interventions during the trial week (Table 2). Therapy sessions were the most common type of co-interventions, including physiotherapy (only utilized by people in groups that did not include telerehabilitation), chiropractic, massage therapy, or GP visit. Number of days of medication use ranged from 14 to 27 equating to 1.3% to 2.5% of person-days (12 participants x 90-day study period = 1080 person-days).

**Table 2:** Adverse events and co-interventions.

Adverse event or co-intervention measure		Advice only	Recommended care	Recommended care and telerehabilitation
**Adverse events, n (%)**				
	Participants reporting adverse event	6 (50)	4 (33)	6 (50)
	Total number of adverse events	10	7	8
	Mild	7 (70)	4 (57)	6 (75)
	Moderate	3 (30)	3 (43)	1 (12)
	Serious	0 (0)	0 (0)	1 (12)
**Co-intervention, n (%)**				
	Participants using co-interventions	2 (17)	2 (17)	2 (17)
	Total number of co-interventions	14	16	15
	Therapy sessions	9 (64)	9 (56)	9 (60)
	Other interventions	1 (7)	5 (31)	3 (20)
	Episodes of medication use	4 (29)	2 (13)	3 (20)
	Participants using medication, n (%)	2 (17)	2 (17)	2 (17)
	Total number of days of medication use^a^	27	14	24

^a^From a total of 1080 person-days (12 participants x 90-day study period).

#### Feasibility of Future Economic Evaluation

The response to health service use questionnaires and iMTA Productivity Cost was acceptable (greater than 80%), indicating it is just as feasible to collect economic outcomes from this population as it is clinical outcomes. Direct costs were Aus $195.64 (SD 224.25; US $137.93), absenteeism was Aus $223.33 (SD 864.41; US $157.45), and presenteeism was Aus $430.23 (SD 752.61; US $303.31) per person across the cohort.

### Secondary clinical outcomes

**Figure 4:**
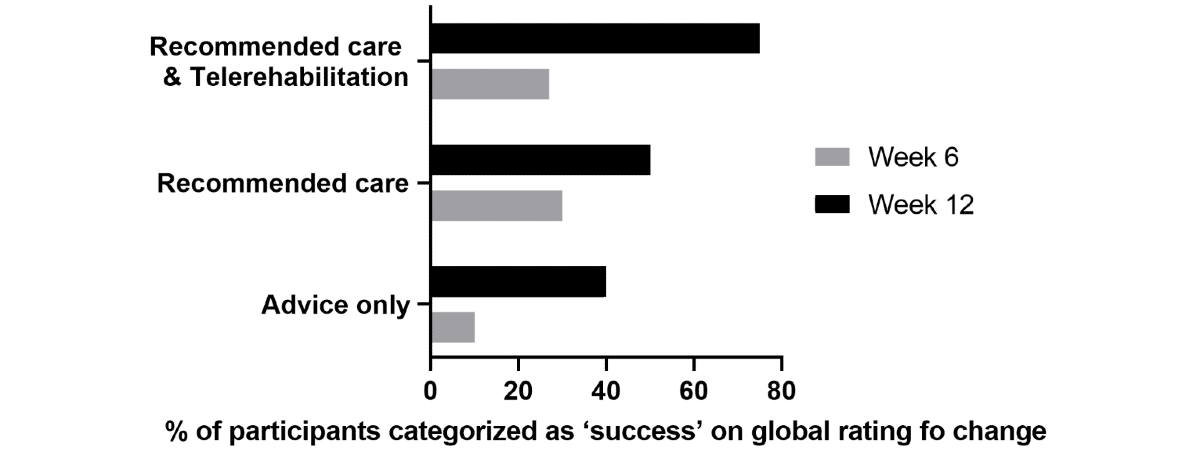
Global rating of change.

Data for secondary outcomes are presented in Figure 4 and Table 3.

**Figure 5:**
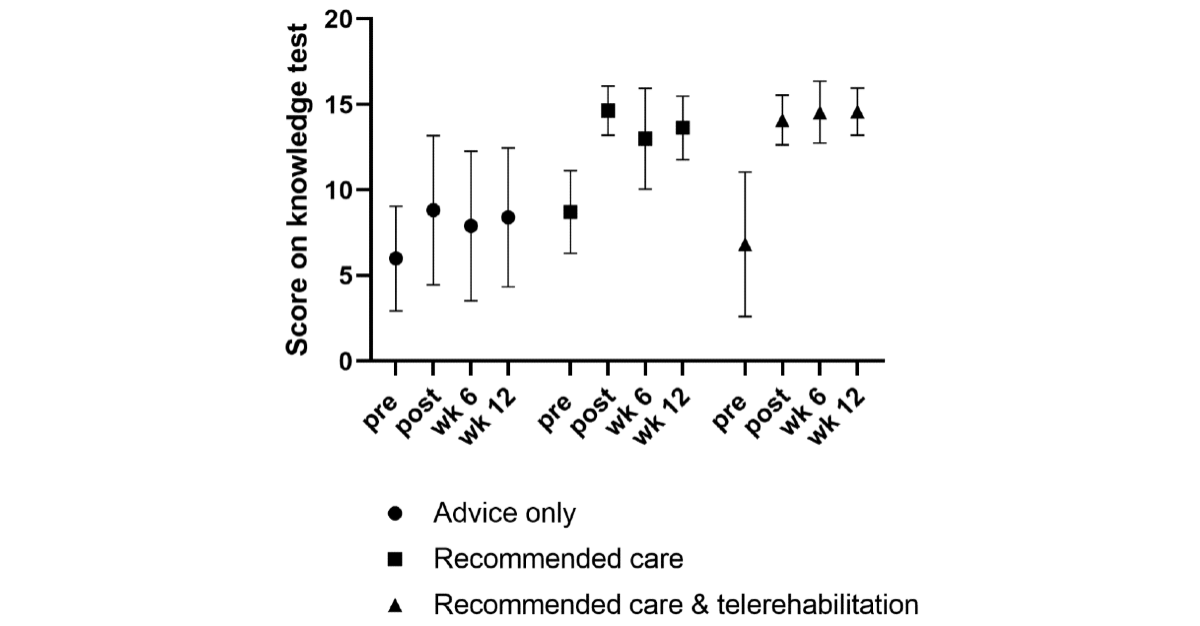
Knowledge test change over time. post: postintervention; pre: preintervention; wk: week.

The median knowledge test score was between 6 and 9 of a possible 16 points on the baseline test (Figure 5). Immediately after exposure to the education intervention, the median increased to 14 or 15 points in the groups receiving the recommended care, with a smaller increase in the advice-only group.

**Table 3:** Secondary outcomes (reported as mean [SD]).

Outcome		Advice only	Recommended care	Recommended care and telerehabilitation
**SPADI^a^**				
	Baseline	30.6 (17.7)	37.3 (16.7)	41.8 (19.1)
	Week 6	32.9 (19.9)	27.3 (18.5)	15.0 (9.5)
	Week 12	26.6 (22.3)	21.7 (17.8)	12.9 (6.9)
	Mean difference at 6 weeks (95% CI)	2.3 (–9.5 to 14.1)	–14.0 (–26.7 to –1.3)	–26.9 (–39.2 to –14.5)
	Mean difference at 12 weeks (95% CI)	–4.8 (–20.3 to 10.8)	–16.0 (–26.0 to –6.0)	–28.9 (–40.9 to –28.7)
	SMD^b^ baseline-week 6	0.1	0.8	1.4
	SMD baseline-week 12	0.3	0.9	1.4
**Worst pain in previous 7 days**				
	Baseline	56.8 (17.9)	51.6 (22.4)	59.7 (21.1)
	Week 6	55.7 (22.2)	41.5 (22.2)	31.9 (23.1)
	Week 12	41.8 (23.1)	44.8 (28.1)	28.1 (25.6)
	Mean difference at 6 weeks (*95*% CI)	–0.2 (–11.3 to 10.9)	–9.3 (–34.9 to 16.29)	–26.8 (–44.8 to –8.9)
	Mean difference at 12 weeks, (95% CI)	–15.8 (–33.1 to 1.5)	–3.2 (–19.7 to 13.4)	–31.6 (–49.89 to –13.28)
	SMD baseline-week 6	0.1	0.6	1.0
	SMD baseline-week 12	0.2	0.6	1.0
**TSK^c^**				
	Baseline	36.0 (7.0)	35.2 (5.8)	35.5 (6.7)
	Week 6	37.3 (6.6)	31.5 (8.9)	32.6 (7.5)
	Week 12	36.3 (6.5)	30.6 (5.7)	31.1 (6.6)
	Mean difference at 6 weeks (95% CI)	–0.2 (–4.2 to 3.8)	–4.8 (–8.8 to –0.8)	–3.6 (–7.2 to 0.1)
	Mean difference at 12 weeks, (95% CI)	–1.9 (–5.9 to 2.1)	–5.0 (–7.0 to –3.0)	–4.4 (–8.8 to –0.1)
	SMD baseline-week 6	0.0	0.6	0.5
	SMD baseline-week 12	0.3	0.8	0.6
**PCS^d^**				
	Baseline	7.3 (9.2)	4.6 (5.8)	4.8 (4.1)
	Week 6	8.8 (10.5)	6.0 (10.5)	3.7 (3.3)
	Week 12	5.1 (0.5 to 9.7)	2.7 (1.1 to 4.2)	4.0 (1.0 to 7.0)
	Mean difference at 6 weeks (95% CI)	0.6 (–1.1 to 2.3)	0.1 (–1.8 to 2.0)	–1.6 (–3.1 to 0.0)
	Mean difference at 12 weeks (95% CI)	–3.4 (–7.3 to 0.5)	–2.0 (–4.6 to 0.6)	–0.8 (–3.3 to 1.6)
	SMD baseline-week 6	0.0	0.0	0.4
	SMD baseline-week 12	0.4	0.5	0.2
**PSEQ^e^**				
	Baseline	50.4 (9.4)	50.1 (9.3)	52.3 (6.9)
	Week 6	49.7 (6.4)	51 (7.6)	55.1 (4.1)
	Week 12	50.5 (7.9)	54.8 (6.5)	55.6 (5.8)
	Mean difference at 6 weeks (95% CI)	–1.1 (–5.1 to 2.9)	1.7 (–7.2 to 10.5)	3.6 (0.8 to 6.3)
	Mean difference at 12 weeks, (95% CI)	1.3 (–4.1 to 6.7)	4.2 (–1.6 to 9.9)	3.3 (1.0 to 5.6)
	SMD baseline-week 6	0.1	0.2	0.6
	SMD baseline-week 12	0.2	0.5	0.5
**EQ5D^f^**				
	Baseline	0.76 (0.11)	0.74 (0.13)	0.74 (0.12)
	Week 6	0.73 (0.12)	0.74 (0.12)	0.77 (0.11)
	Week 12	0.73 (0.09)	0.77 (0.13)	0.78 (0.07)
	Mean difference at 6 weeks (95% CI)	–0.02 (–0.10 to 0.06)	0.02 (–0.08 to 0.12)	0.05 (–0.02 to 0.12)
	Mean difference at 12–weeks, (95% CI)	–0.02 (–0.14 to 0.10)	0.01 (–0.10 to 0.12)	0.03 (–0.02 to 0.09)
	SMD baseline-week 6	0.2	0.2	0.4
	SMD baseline-week 12	0.2	0.1	0.3

^a^SPADI: shoulder pain and disability index.

^b^SMD: standardized mean difference.

^c^TSK: Tampa Scale for Kinesiophobia.

^d^PCS: Pain Catastrophizing Scale.

^e^PSEQ: Pain Self-Efficacy Questionnaire.

^f^EQ5D: EuroQol 5D-5L.

## Discussion

### Principal Findings

Based on our predefined progression criteria, conducting a full-scale RCT to evaluate delivery of care via the internet and telerehabilitation for RCRSP is feasible. Prespecified rates of conversion (36/38, 96%; criterion of greater than 20%), recruitment (36 per month), and retention (34/36, 94%; criterion of greater than 80%) were high, and there were acceptable responses to questionnaire outcomes at all time points (30/36, 83% or higher; criterion of greater than 80%). Adherence was acceptable (2 to 3 sessions per week among more than 70% of participants) for the group receiving recommended care with telerehabilitation (11/12, 92%) but not for the group receiving recommended care without telerehabilitation (8/12, 67%). The trial was not powered to investigate between-group differences, but we have reported descriptive variability and within-group change data for clinical outcomes in accordance with pilot trial recommendations [60].

Although recruitment was efficient using our social media strategy, there was a gender imbalance favoring women (32/36, 89%). A systematic review of health behavior change interventions also reported much higher participation among women even though both genders were targeted via a social media campaign [61]. Addressing gender balance is an important consideration for a future full-scale trial to ensure that the findings are generalizable to the population with RCRSP. There are reports that men are more likely to respond to Facebook ads that utilize concise text and appeal to leadership themes and masculine themes, and less likely to respond to unisex ads [62]. Facebook also enables targeting ads only to men and this could be used during a full-scale trial if gender imbalance emerged.

By removing cost and travel barriers, our internet and remote interventions may have promoted health-seeking behavior among people who would not ordinarily seek care. Our participants were slightly younger (mean age 54 years versus 60 years) and had slightly lower SPADI scores (37 versus 43) compared with a large, high-quality trial that recruited people with RCRSP via primary care from the same geographic region [49]. However, more people in our trial had tried exercise or physiotherapy treatment (69% versus 38%) [49], indicating that most of our cohort is representative of people who seek care for RCRSP. Nevertheless, strategies to increase the RCRSP severity of people recruited (eg, a minimum SPADI score as an inclusion criterion) may increase the extent to which our cohort is representative of people who seek care for this condition in primary care.

The Zelen randomization design that we used (theoretically) provides two key design advantages: (1) it improves recruitment rates by avoiding consent refusal related to simple randomization, and (2) it reduces attrition (and therefore selection bias) related to resentful demoralization among people in the control group [63]. This approach may have contributed to the efficiency of recruitment and high retention in this feasibility trial. Opting to remain in the original group rather than joining the group that people are randomized to (also referred to as crossover) is another potential issue with the Zelen design [63], but 100% of people accepted the offer of randomization in our trial. A potential limitation of the Zelen design is that people offered additional treatment perceive this as superior to the original care they consented to, but this can be mitigated by careful wording of the trial information to introduce uncertainty about the comparative efficacy of the interventions being tested. Overall, we recommend that researchers consider Zelen’s design for internet-based interventions where recruitment and retention may be challenging (eg, where there is no human contact).

Efficient recruitment and high retention rates may also be related to the monetary incentive (Aus $100 [US $70] for 12-week outcomes). Previous systematic reviews have reported that monetary incentives can improve retention rates for studies that utilise postal or electronic questionnaires [33-35]. We employed this strategy because of the substantial attrition rates that have been reported in some trials investigating internet interventions aimed at improving health outcomes, including interventions for musculoskeletal conditions [26,64,65]. For example, 45% attrition was reported at 3 months for an internet-delivered physical activity intervention for knee OA [66]. Although we have no knowledge of rates of attrition without the monetary incentive, attrition may be an issue for our interventions, particularly the advice-only intervention (active control).

Contact with a physiotherapist may explain the higher exercise adherence in the group receiving telerehabilitation. Consistent with our findings, there are reports of low adherence for internet-only interventions [67], and a recent study found that website-based exercise with remote physiotherapist support improved adherence (at 4 weeks) compared with the provision of a leaflet explaining the exercises [68]. Participants in our study may have perceived value in telerehabilitation (eg, improved explanation, reassurance, feedback, monitoring, individualization, and peer support). Alternatively, our findings may be explained by Hawthorne effects [69]. We used behavioral strategies to improve adherence in the internet intervention (eg, electronic exercise diary, addressing beliefs), although “persuasive technologies” such as electronic reminders or tailoring to individual users’ needs or personalities may further improve adherence in the group receiving recommended care without telerehabilitation [70].

### Comparison With Prior Work

There are numerous internet-only delivery of care interventions targeted toward people with chronic pain as well as OA, but feasibility (retention and adherence) and outcomes have been mixed [26,66,71]. There is also evidence that care for people with musculoskeletal conditions can be successfully delivered via telerehabilitation. Cottrell et al [21] reviewed trials investigating telerehabilitation interventions for musculoskeletal conditions (knee OA, neck pain) and after shoulder and knee joint surgery. The review concluded that the telerehabilitation-only interventions, which are comparable with our recommended care and telerehabilitation group, were equivalent to face-to-face care for function outcomes. Our intervention is novel in that it blends internet, telerehabilitation, and group-based care delivery for RCRSP.

### Strengths

The content of our education intervention was informed by stakeholders, including patients, clinicians, or researchers with expertise in shoulder pain management [42]. Stakeholders helped to determine the education needs for people with RCRSP and subsequently assessed accuracy and clarity of the content and delivery modes. The exercises were based on consensus statements [37,38], systematic reviews [36,39-41], practice guidelines [5-7], and a protocol for a high-quality trial currently underway in the United Kingdom [72]. The intervention incorporated only two exercises to ensure simplicity for the internet-only delivery mode and included behavioral strategies to increase exercise adherence [72].

### Limitations

This trial design has limitations. First, generalizability to a primary care population may be improved by implementing strategies to improve the gender balance and recruit people with greater RCRSP severity (as discussed above). Second, we did not measure adherence to recommendations to modify activity behavior (the only intervention component in the advice-only group), which may help to explain findings in this group. Third, the findings may be influenced by placebo and contextual factors given that participants in the intervention groups were aware that they were allocated to an intervention involving additional evidence-based care. In our pragmatic trial design, we chose a control intervention that represents an acceptable standard of care [73]. Fourth, diagnosis was based on remote screening (online and teleconference). Although it was based on guidelines [7], this remote method has not been tested.

### Conclusion

Our prespecified success criteria were met or exceeded but there was a gender imbalance toward women. It is feasible to progress to a fully powered trial, but strategies to address the gender imbalance need to be implemented.

## Appendix

Multimedia Appendix 1 Educational materials for advice-only group.

Multimedia Appendix 2 Activity-modification guide for advice-only group.

Multimedia Appendix 3 Educational materials for recommended care group.

Multimedia Appendix 4 Progression and regression criteria for exercise.

Multimedia Appendix 5 Website interface of exercise progression and regression tool with built-in electronic exercise diary.

## Abbreviations

Aus $: Australian dollars
CERT: Consensus on Exercise Recording Template
CONSORT: Consolidated Standards of Reporting Trials
EQ5D: EuroQol 5D-5L
GP: general practitioner
GROC: Global Rating of Change
iPCQ: iMTA Productivity Cost Questionnaire
MCID: minimal clinically important difference
OA: osteoarthritis
PCS: Pain Catastrophizing Scale
PEMAT: Patient Education Materials Assessment Tool
PSEQ: Pain Self-Efficacy Questionnaire
RCRSP: rotator cuff–related shoulder pain
RCT: randomized controlled trial
SMD: standardized mean difference
SPADI: shoulder pain and disability index
TSK: Tampa Scale for Kinesiophobia
VAS: visual analog scale
