# Supplementary material for: Internet and Telerehabilitation-Delivered Management of Rotator Cuff–Related Shoulder Pain (INTEL Trial): Randomized Controlled Pilot and Feasibility Trial
Source: JMIR Mhealth Uhealth. 2020 Nov 18;8(11):e24311. doi: 10.2196/24311 (PMC7710452; doi:10.2196/24311)
Supplement: Multimedia Appendix 1 [file mhealth_v8i11e24311_app1.pdf]

ROTATOR CUFF RELATED PAIN EDUCATION

+ Shoulder pain is common
+ Rotator cuff-related pain is the most common cause
+ For most people it will improve over time with advice and exercise

The shoulder has two joints:

- The glenohumeral joint between the arm bone (humerus) and shoulder blade (scapula). It is a ball and socket joint.
- The acromioclavicular (AC joint) between the top of the shoulder blade (acromion) and collar bone (clavicle)

What is the "rotator cuff"?

- The rotator cuff is a group of muscles coming from the shoulder blade and wraps around the ball and socket joint.
- The rotator cuff muscles produce movement at the shoulder (i.e. when lifting your arm) and help to keep the ball nicely centred in the socket when the arm moves
- There is a bursa (fluid filled sack) above the rotator cuff tendons that lubricates movement between the tendons and the bone above (acromion)

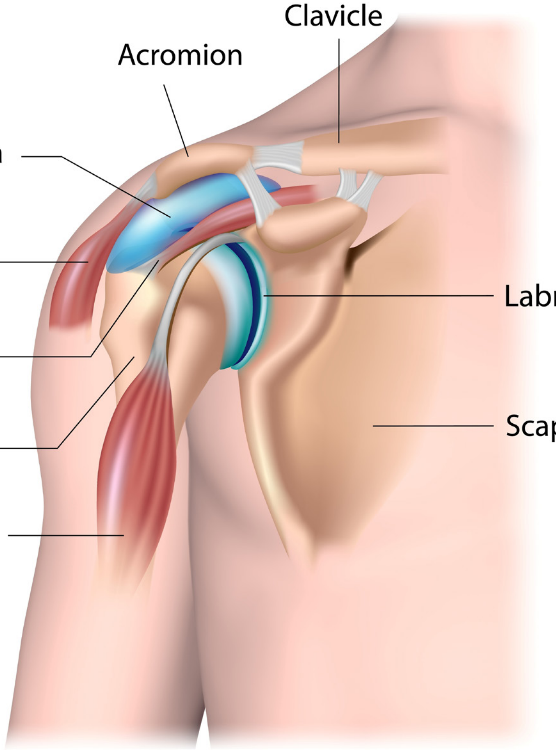

MONASH University

What causes rotator cuff related pain?

ROTATOR CUFF RELATED PAIN IS COMPLEX AND MAY INVOLVE A COMBINATION OF PHYSICAL AND LIFESTYLE RELATED FACTORS.

Repetitive overhead arm movements are often the main cause, especially an increase in the amount of these movements. Common examples of this include:

- Starting a new exercise program or sport that involves shoulder activity
- Returning to activity too fast after time off due to illness or injury
- Taking on a new job that involves repetitive overhead activity

As shown in this image, many other factors can make you more susceptible to developing rotator cuff related pain

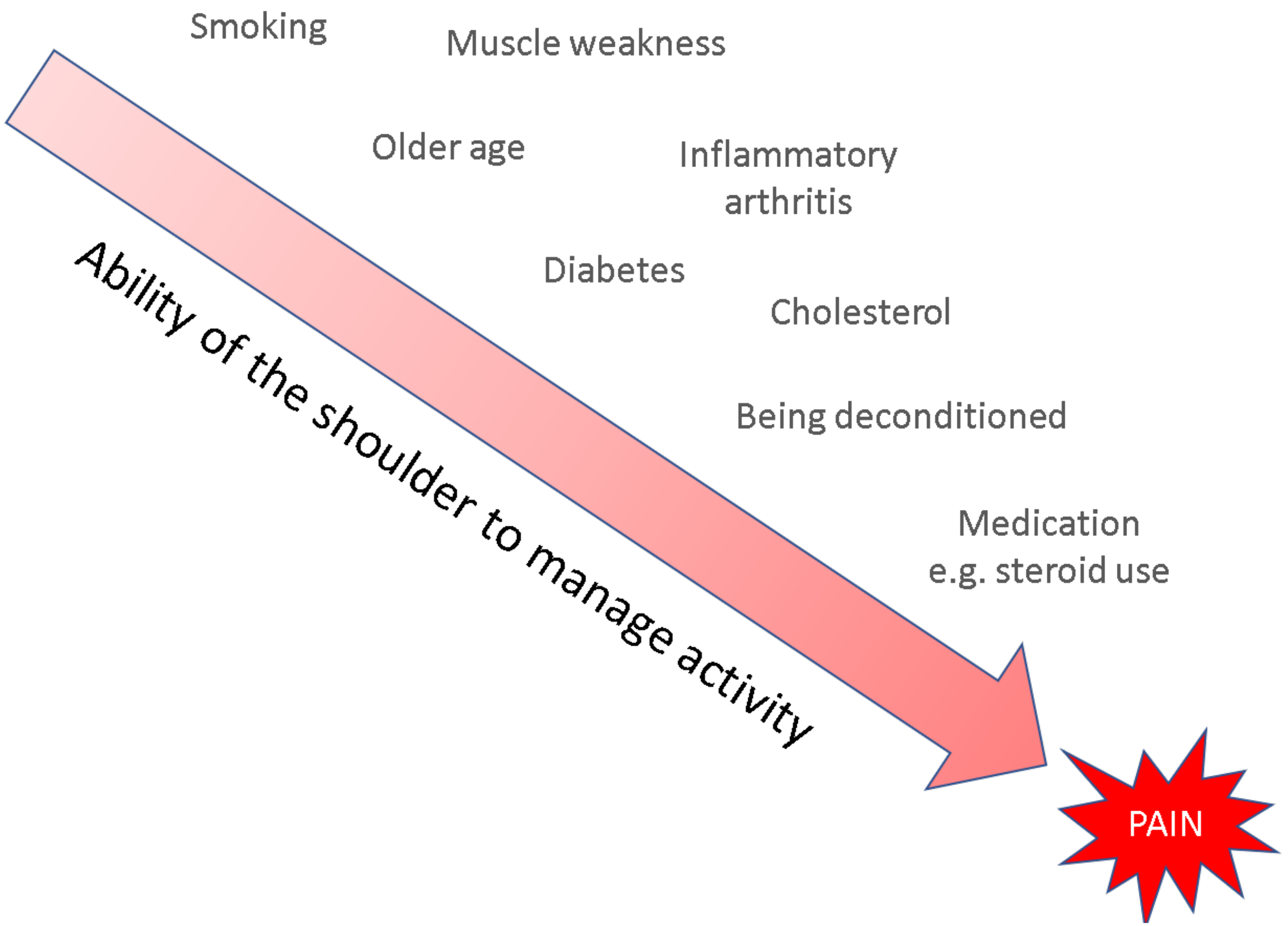

Most rotator cuff related pain comes on gradually without trauma, but the rotator cuff can also be injured after a sudden traumatic event such as a fall. If your pain came on after a fall or other trauma, it is important to see your doctor as there may be an injury requiring medical attention

MONASH University
