# Supplementary material for: Internet and Telerehabilitation-Delivered Management of Rotator Cuff–Related Shoulder Pain (INTEL Trial): Randomized Controlled Pilot and Feasibility Trial
Source: JMIR Mhealth Uhealth. 2020 Nov 18;8(11):e24311. doi: 10.2196/24311 (PMC7710452; doi:10.2196/24311)
Supplement: Multimedia Appendix 2 [file mhealth_v8i11e24311_app2.pdf]

But it hurts!

IS IT OK TO FEEL SOME PAIN DURING ACTIVITY AND EXERCISES THAT INVOLVES MY SHOULDER?

Yes. It is common and in fact quite normal to feel some pain when performing or after performing activity or exercise, provided that the pain settles within a reasonable period of time.

Some pain during activity does not mean you are doing any damage.

PAIN ≠ DAMAGE

IF YOU STOP USING YOUR SHOULDER BECAUSE OF PAIN, IT IS LIKELY YOUR SHOULDER WILL BECOME WORSE OVER THE LONG TERM. AS SUCH IT IS VERY IMPORTANT TO CONTINUING USING YOUR SHOULDER IN YOUR DAILY ACTIVITIES AS MUCH AS PAIN ALLOWS

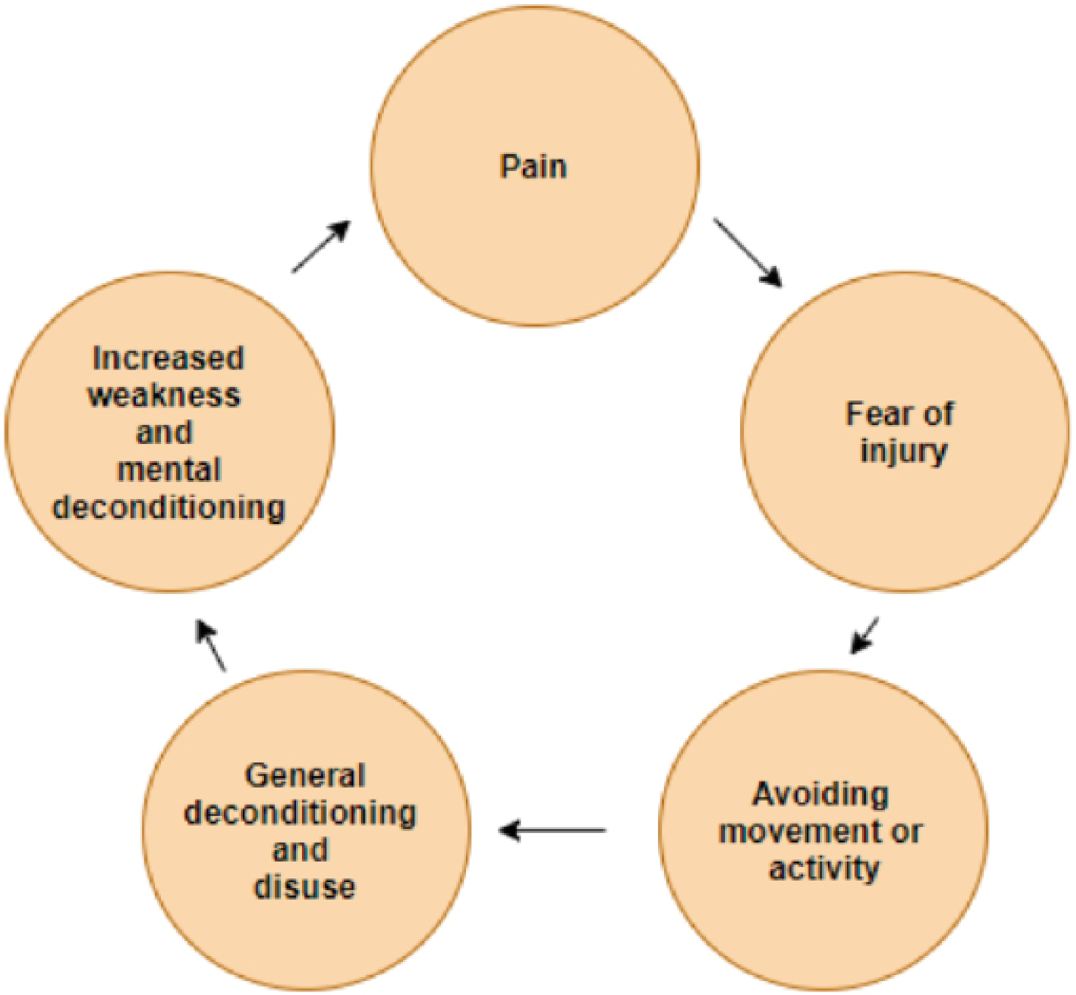

How much pain during movement is acceptable?

The Visual Analogue Scale for pain shown below measures self-reported pain between zero - no pain at all and 10 - the worst pain

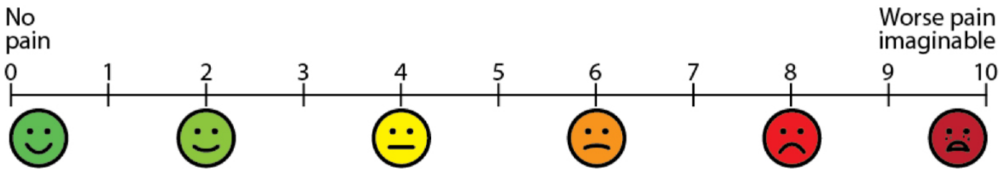

'Acceptable pain' is pain that you define as 4/10 or less.

Modify movements and activities that may provoke your pain

If you feel an activity or exercise is too painful, stop doing it or modify how you do it. Modify or stop activities that provoke pain that is 5/10 or more. Common activities and potential modifications are shown in the figures above

Activity	Potential Modification
**Hanging washing on the line**	Hanging washing on a clothes horse at waist height
**Clasping a bra at back**	Do up bra at front, then twist to the back
**Lifting groceries onto high shelf**	Organise commonly used grocery items to be at waist level
**Swimming freestyle**	Modify to breaststroke or survival backstroke
**Lying on your affected side**	Sleep on your back or unaffected side
**Work-related above head tasks**	Modify work environment or activities
**Putting a jumper on above head**	Wear a top that does up with a zip or button
**Reaching into the back seat of your car**	Position items in the front seat, or on the rear floor rather than rear seat so you are not reaching as high
**Housework involving vacuuming and scrubbing**	Reduce the time that you spend doing these activities in the short term
**Overhead shoulder exercises in the gym**	Reduce the weight or don't lift your arm as high above your head

MONASH University

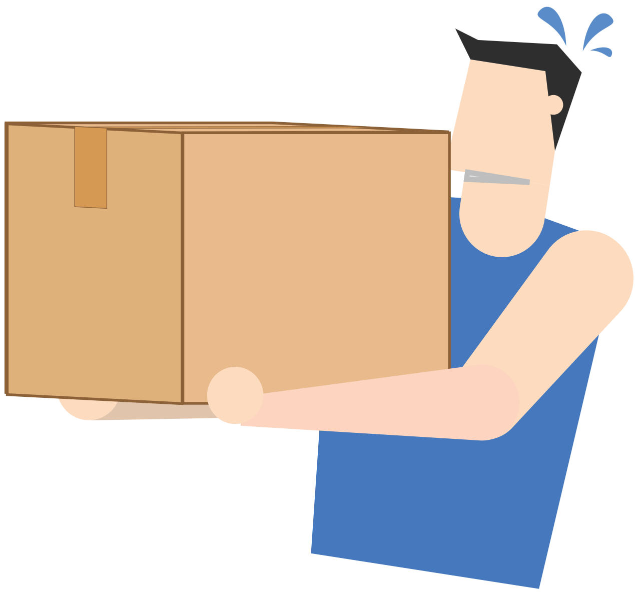

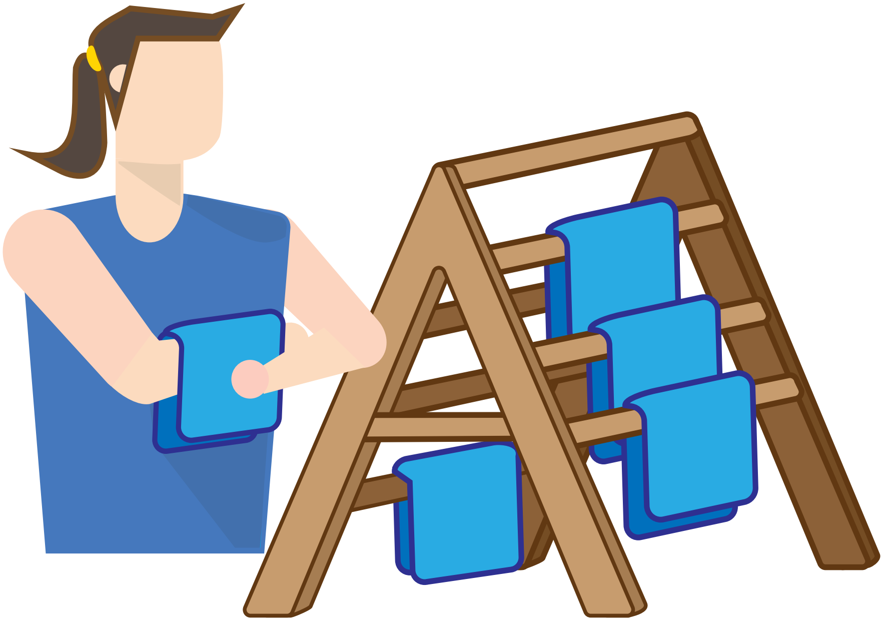

Gradually resume movements and activities when they are less painful

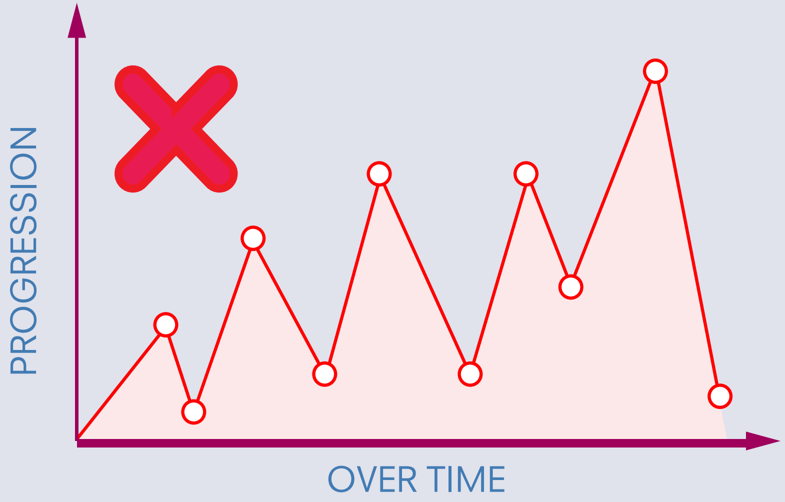

When your pain settles again, it is safe and suggested to recommence the in household / everyday activities or exercises, but you need to ensure that activity is introduced gradually.

Sudden changes in activity can cause your symptoms to worsen

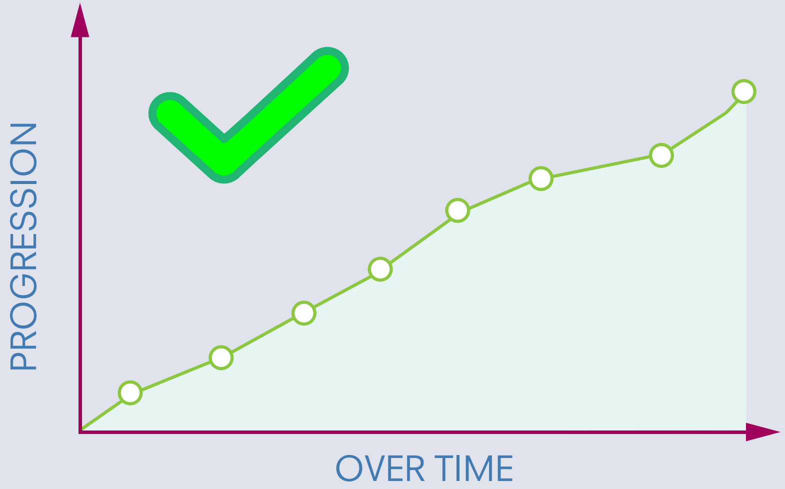

MONASH University

WHERE CAN I SOURCE MORE INFORMATION?

MONASH University

We have provided additional information below in Appendix 1 if you would like additional information or detail. We also recommend the following online resources:

Better Health Channel

Choosing Wisely Australia

National Prescribing Service

Medicine Line 1300 633 424

ACTIVITY MODIFICATION PLANNER

MONASH University

THE FIRST STEP IS TO IDENTIFY AND THEN MODIFY THESE ACTIVITIES. USE THE TABLE BELOW TO WRITE DOWN ACTIVITIES AND THE WAY YOU WILL MODIFY THEM. PRINT THIS PAGE OUT AND STICK IT ON YOUR FRIDGE OR BATHROOM MIRROR TO REMIND YOU

Track your progress

You will complete surveys at Week 6 and Week 12 of the program. This will track the effect of the above changes on your shoulder pain and function.

Activity	Modification	Pain with original activity	Pain with modification
Example			
Putting a jumper on above head	Wear a jacket with a zip or a shirt with buttons instead	8/10	4/10

Once you have completed the 12-week program (and all outcome measures), you will recieve a $100 Shopping Voucher.
