# Supplementary material for: Internet and Telerehabilitation-Delivered Management of Rotator Cuff–Related Shoulder Pain (INTEL Trial): Randomized Controlled Pilot and Feasibility Trial
Source: JMIR Mhealth Uhealth. 2020 Nov 18;8(11):e24311. doi: 10.2196/24311 (PMC7710452; doi:10.2196/24311)
Supplement: Multimedia Appendix 3 [file mhealth_v8i11e24311_app3.pdf]

ROTATOR CUFF RELATED PAIN EDUCATION

+ Shoulder pain is common
+ Rotator cuff-related pain is the most common cause
+ For most people it will improve over time with advice and exercise

The shoulder has two joints:

- The glenohumeral joint between the arm bone (humerus) and shoulder blade (scapula). It is a ball and socket joint.
- The acromioclavicular (AC joint) between the top of the shoulder blade (acromion) and collar bone (clavicle)

What is the "rotator cuff"?

- The rotator cuff is a group of muscles coming from the shoulder blade and wraps around the ball and socket joint.
- The rotator cuff muscles produce movement at the shoulder (i.e. when lifting your arm) and help to keep the ball nicely centred in the socket when the arm moves
- There is a bursa (fluid filled sack) above the rotator cuff tendons that lubricates movement between the tendons and the bone above (acromion)

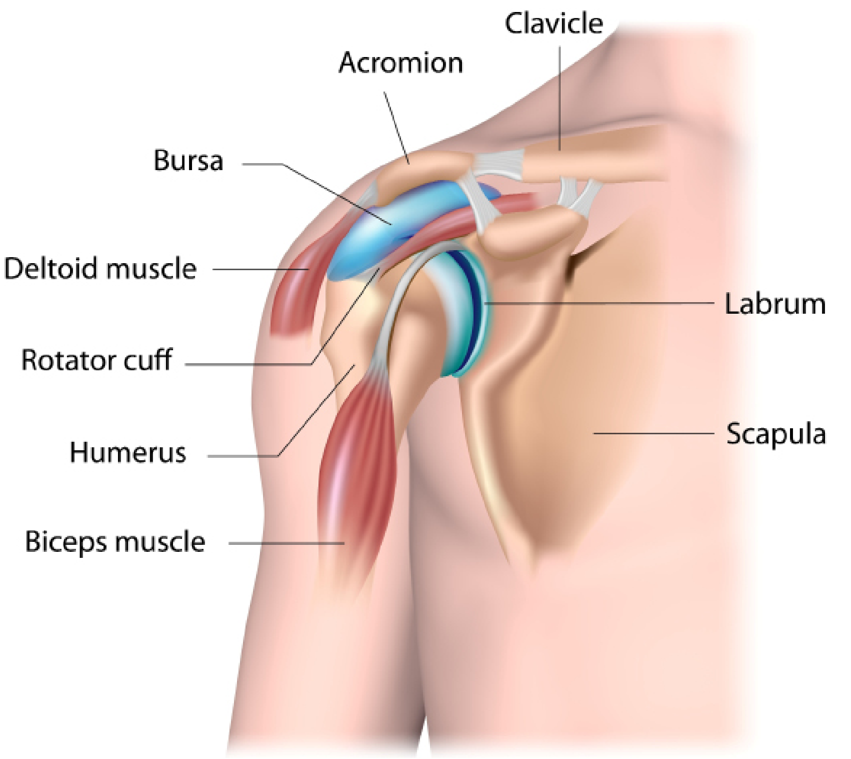

MONASH University

What causes rotator cuff related pain?

ROTATOR CUFF RELATED PAIN IS COMPLEX AND MAY INVOLVE A COMBINATION OF PHYSICAL AND LIFESTYLE RELATED FACTORS.

Repetitive overhead arm movements are often the main cause, especially an increase in the amount of these movements. Common examples of this include:

- Starting a new exercise program or sport that involves shoulder activity
- Returning to activity too fast after time off due to illness or injury
- Taking on a new job that involves repetitive overhead activity

As shown in this image, many other factors can make you more susceptible to developing rotator cuff related pain

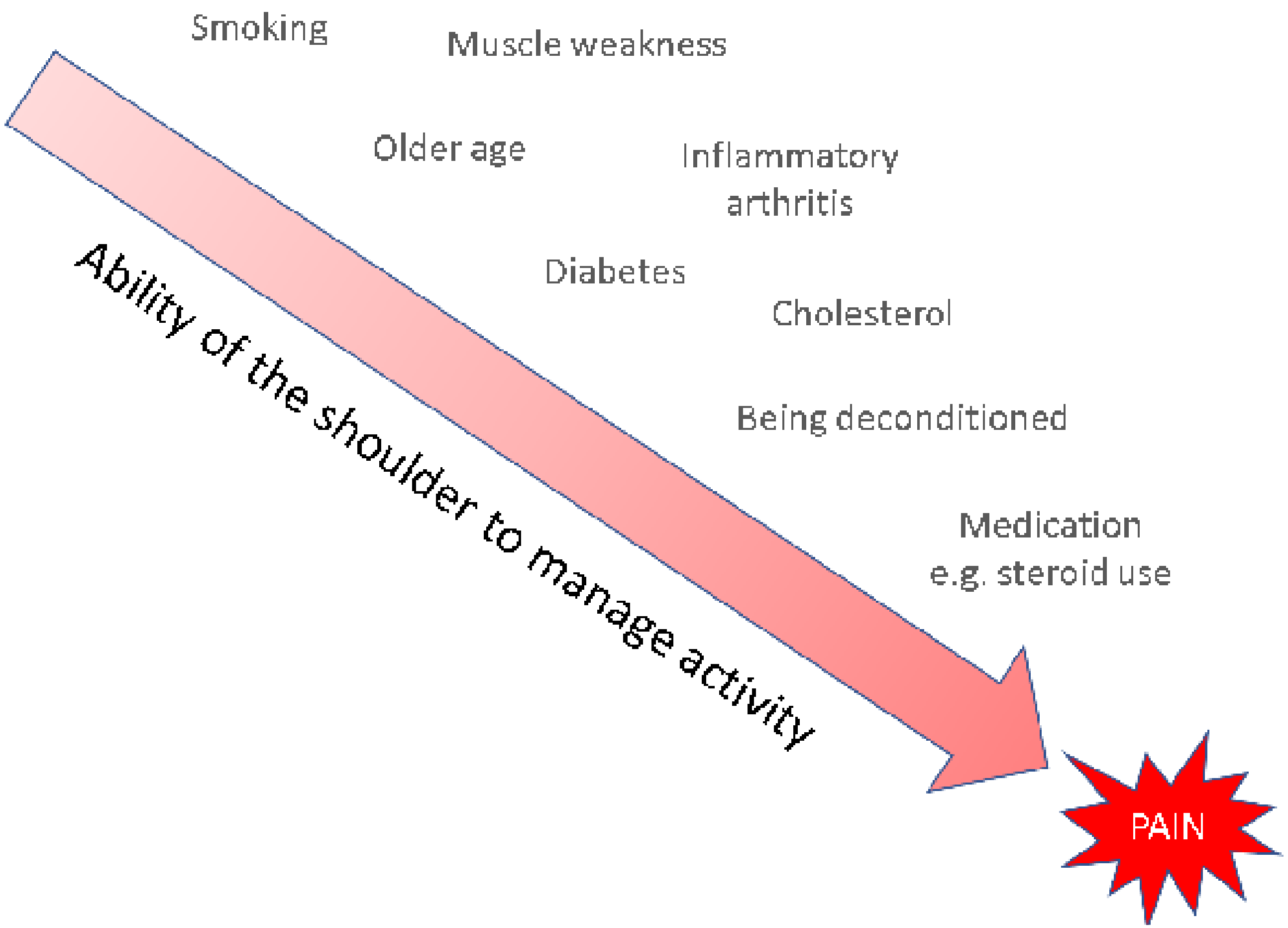

Most rotator cuff related pain comes on gradually without trauma, but the rotator cuff can also be injured after a sudden traumatic event such as a fall. If your pain came on after a fall or other trauma, it is important to see your doctor as there may be an injury requiring medical attention

MONASH University

What are the most common symptoms of rotator cuff related pain?

- Pain at the front and side of the shoulder: often evident at night when lying on your side.
- Pain when lifting the arm above shoulder height
- The range of movement in the shoulder is usually not impacted by stiffness; but may be painful.

How is rotator cuff related pain diagnosed?

Rotator cuff related pain can be diagnosed by health care professionals who treat shoulder pain such as a sports doctor, general practitioner, physiotherapist or osteopath. Diagnosis is based on asking questions about what aggravates your shoulder pain and conducting an examination including testing your shoulder movement and strength.

MONASH University

DO I NEED A SCAN?

NO. A SCAN LIKE AN MRI OR ULTRASOUND IS NOT REQUIRED TO CONFIRM ROTATOR CUFF-RELATED PAIN

It is important to know that what is shown on a scan does not always relate to pain. It is very normal to see changes of rotator cuff tendons on scans, even in people with no pain at all! Common findings include partial or full thickness tendon tears and thickening of the bursa around the tendons. These changes can occur naturally in most people over a passage of time as a result of age and activity. Because of this inconsistency between scans and pain, it is essential that your pain is diagnosed via a clinical examination.

If people are not responding to treatment a scan would usually be indicated after around twelve weeks.

DO I NEED SURGERY?

MOST PEOPLE WITH THIS TYPE OF SHOULDER PAIN DO NOT REQUIRE SURGERY

Many people logically believe that if they have a tear in their tendon, it needs to be repaired by having surgery.

However, we know that around 80% of people respond to advice and exercise, even people with full thickness rotator cuff tendon tears.

Surgery is only considered when someone has not responded to adequate exercise and education for 12 weeks or longer.

What does surgery involve and what are the benefits?

There are two main surgeries that may be considered if all other conservative treatments have failed.

SUBACROMIAL DECOMPRESSION

It is thought that tissues under the acromion can be 'impinged' or compressed by the bone. In this surgery part of the acromion bone is removed to relieve these tissues. Unfortunately, research has shown that people who have this surgery have similar outcomes compared to people who have placebo surgery

ROTATOR CUFF TENDON REPAIR

This surgery involves the surgical repair of damaged or torn rotator cuff tendons. It has been shown that around 40% of rotator cuff repairs re-tear or fail to heal post-surgery. Improvement in symptoms post-surgery may be a result of the placebo effect or adherence to post-operative physiotherapy, instead of the actual surgery itself.

What are potential harms of surgery?

These can include complications occurring during (intraoperative) or after surgery and can include infection, blood clots, cardiac arrest, allergic reaction to medication or even death. These complications are rare but serious. There is also a possibility that your shoulder pain and function will not improve post-surgery.

MONASH University

What are the alternatives to surgery?

Guidelines recommend that a trial of non-surgical treatment for 12 weeks. Exercise and advice at the bottom of the pyramid is the most recommended treatment, and this involves

- A tailored and progressive exercise program to improve function
- Avoiding or modifying very painful activities in the short-term
- Taking analgesia or anti-inflammatory medications.

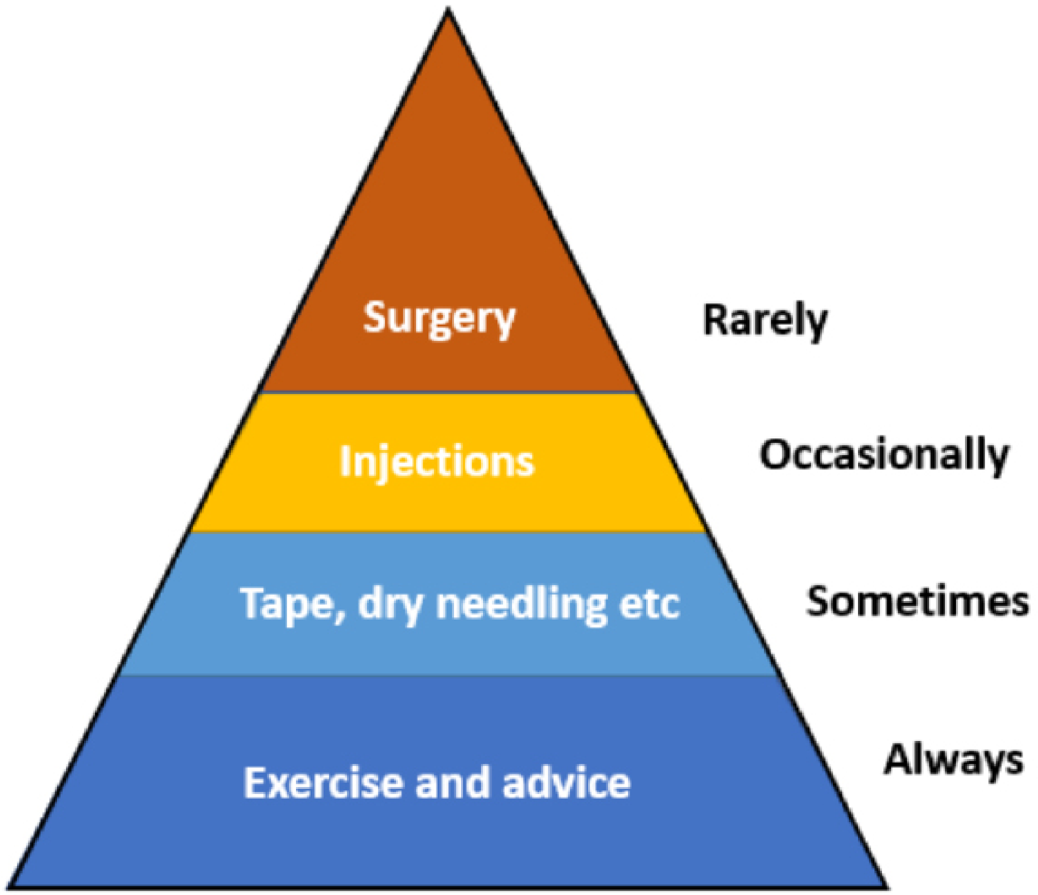

Other interventions like taping, dry needling or massage may be helpful for some people.

Injections can be utilised to reduce pain and allow a person to complete their exercises.

What are the benefits of exercise and advice?

INTRODUCING EXERCISE GRADUALLY IS CAN INCREASE YOUR SHOULDER STRENGTH AND CAPACITY.

There have not been enough high quality studies comparing advice and exercise to placebo. However, we do know that a majority of people who trial adequate exercise and advice recover without requiring surgery.

What are potential harms associated with exercise for my shoulder?

Potential harms include short-term increase in pain or some muscle soreness. Exercise is highly unlikely to make the tissue changes in your shoulder any worse

How long will it take to feel better?

MOST PEOPLE EXPERIENCE SIGNIFICANT IMPROVEMENT IN THEIR SHOULDER PAIN AND ABILITY TO PERFORM THEIR NORMAL ACTIVITIES WITH THE RIGHT ADVICE AND EXERCISE OVER A PERIOD OF 12 WEEKS

MONASH University

But it hurts!

IS IT OK TO FEEL SOME PAIN DURING ACTIVITY AND EXERCISES THAT INVOLVES MY SHOULDER?

Yes. It is common and in fact quite normal to feel some pain when performing or after performing activity or exercise, provided that the pain settles within a reasonable period of time.

Some pain during activity does not mean you are doing any damage.

PAIN ≠ DAMAGE

IF YOU STOP USING YOUR SHOULDER BECAUSE OF PAIN, IT IS LIKELY YOUR SHOULDER WILL BECOME WORSE OVER THE LONG TERM. AS SUCH IT IS VERY IMPORTANT TO CONTINUING USING YOUR SHOULDER IN YOUR DAILY ACTIVITIES AS MUCH AS PAIN ALLOWS

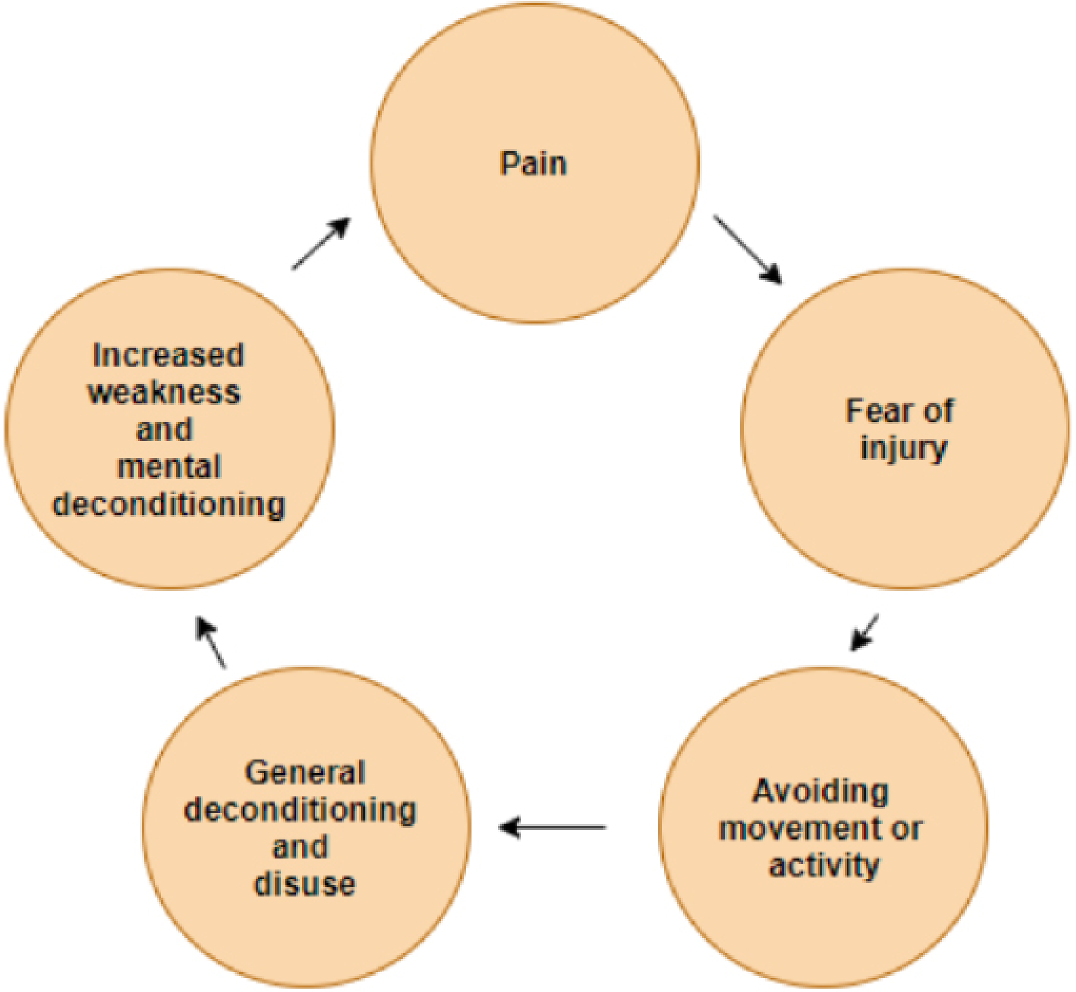

How much pain during movement is acceptable?

The Visual Analogue Scale for pain shown below measures self-reported pain between zero - no pain at all and 10 - the worst pain

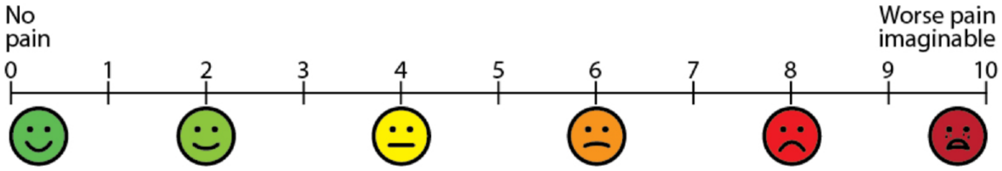

'Acceptable pain' is pain that you define as 4/10 or less.

Modify movements and activities that may provoke your pain

If you feel an activity or exercise is too painful, stop doing it or modify how you do it. Modify or stop activities that provoke pain that is 5/10 or more. Common activities and potential modifications are shown in the figures above

Activity	Potential Modification
**Hanging washing on the line**	Hanging washing on a clothes horse at waist height
**Clasping a bra at back**	Do up bra at front, then twist to the back
**Lifting groceries onto high shelf**	Organise commonly used grocery items to be at waist level
**Swimming freestyle**	Modify to breaststroke or survival backstroke
**Lying on your affected side**	Sleep on your back or unaffected side
**Work-related above head tasks**	Modify work environment or activities
**Putting a jumper on above head**	Wear a top that does up with a zip or button
**Reaching into the back seat of your car**	Position items in the front seat, or on the rear floor rather than rear seat so you are not reaching as high
**Housework involving vacuuming and scrubbing**	Reduce the time that you spend doing these activities in the short term
**Overhead shoulder exercises in the gym**	Reduce the weight or don't lift your arm as high above your head

MONASH University

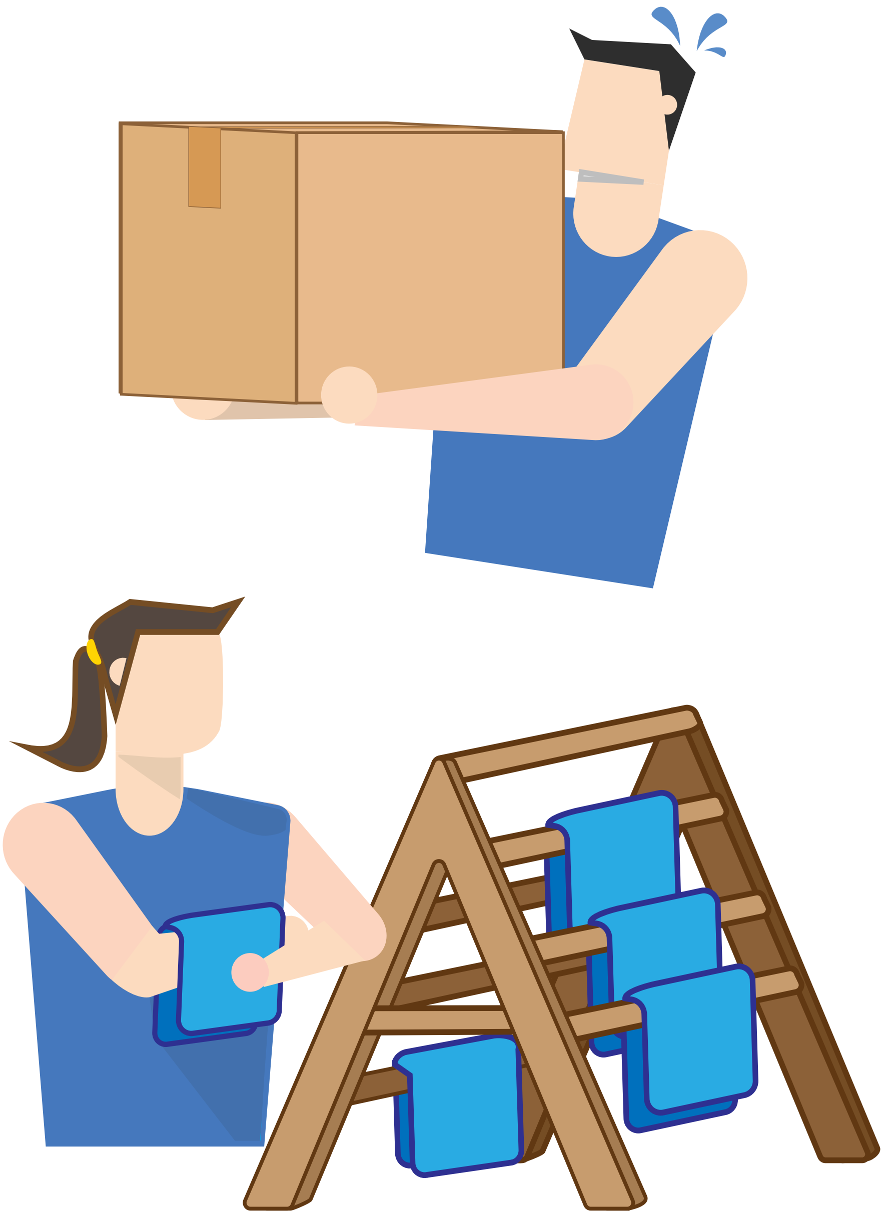

Gradually resume movements and activities when they are less painful

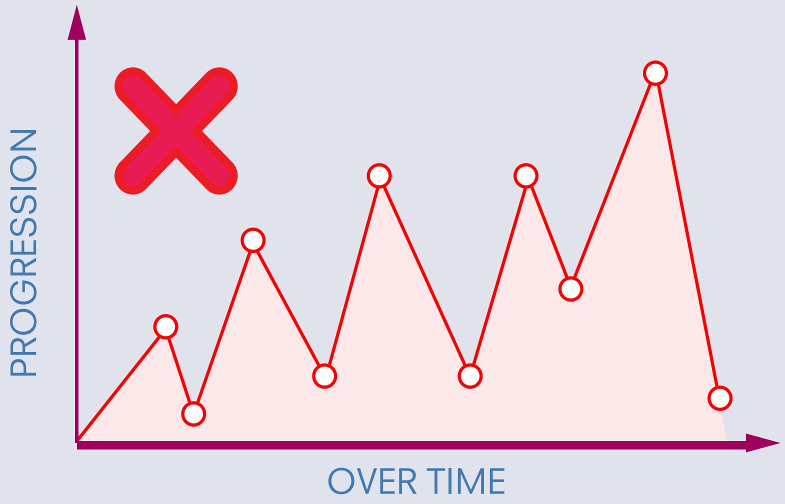

When your pain settles again, it is safe and suggested to recommence the in household / everyday activities or exercises, but you need to ensure that activity is introduced gradually.

Sudden changes in activity can cause your symptoms to worsen

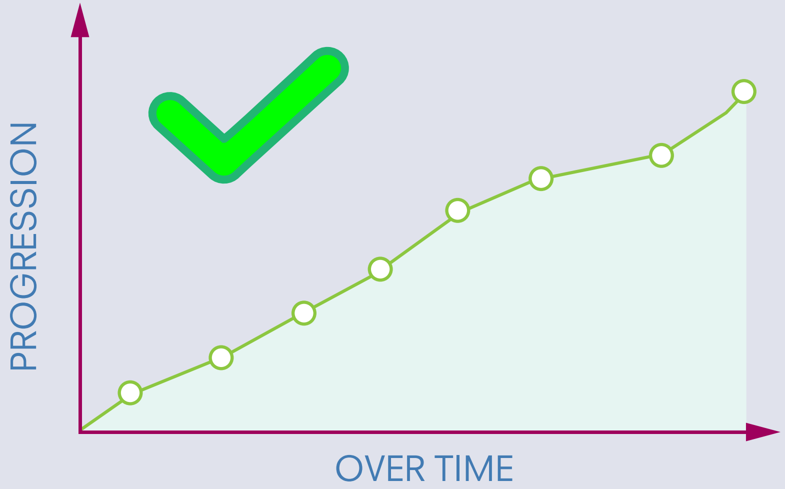

MONASH University

WHERE CAN I SOURCE MORE INFORMATION?

MONASH University

We have provided additional information below in Appendix 1 if you would like additional information or detail. We also recommend the following online resources:

Better Health Channel

Choosing Wisely Australia

National Prescribing Service

Medicine Line 1300 633 424

ACTIVITY MODIFICATION PLANNER

THE FIRST STEP IS TO IDENTIFY AND THEN MODIFY THESE ACTIVITIES. USE THE TABLE BELOW TO WRITE DOWN ACTIVITIES AND THE WAY YOU WILL MODIFY THEM. PRINT THIS PAGE OUT AND STICK IT ON YOUR FRIDGE OR BATHROOM MIRROR TO REMIND YOU

MONASH University

Track your progress

You will complete surveys at Week 6 and Week 12 of the program. This will track the effect of the above changes on your shoulder pain and function.

Activity	Modification	Pain with original activity	Pain with modification
Example			
Putting a jumper on above head	Wear a jacket with a zip or a shirt with buttons instead	8/10	4/10

Once you have completed the 12-week program (and all outcome measures), you will recieve a $100 Shopping Voucher.
