# Supplementary material for: Internet and Telerehabilitation-Delivered Management of Rotator Cuff–Related Shoulder Pain (INTEL Trial): Randomized Controlled Pilot and Feasibility Trial
Source: JMIR Mhealth Uhealth. 2020 Nov 18;8(11):e24311. doi: 10.2196/24311 (PMC7710452; doi:10.2196/24311)
Supplement: Multimedia Appendix 4 [file mhealth_v8i11e24311_app4.pdf]

MODIFICATIONS

THE EXERCISE IS TOO HARD

Follow the arrows in the light blue background

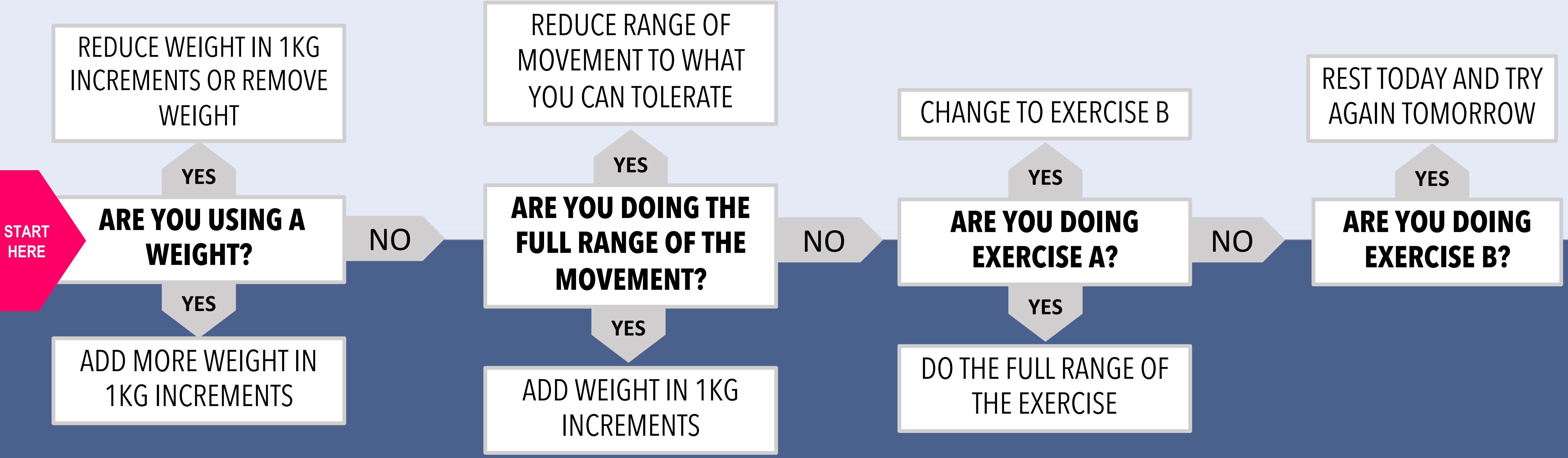

Follow the arrows in the dark blue background

THE EXERCISE IS TOO EASY
