# Supplementary figures and images for: Internet and Telerehabilitation-Delivered Management of Rotator Cuff–Related Shoulder Pain (INTEL Trial): Randomized Controlled Pilot and Feasibility Trial
Source: JMIR Mhealth Uhealth. 2020 Nov 18;8(11):e24311. doi: 10.2196/24311 (PMC7710452; doi:10.2196/24311)

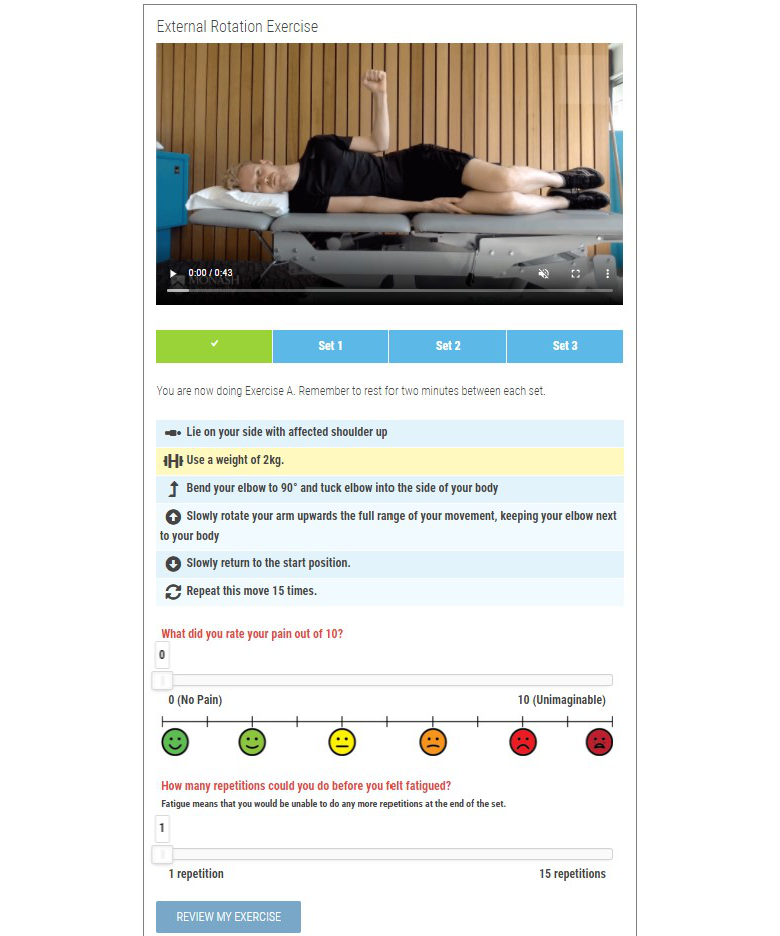

Supplement: Multimedia Appendix 5 [file mhealth_v8i11e24311_app5.png]
